# Complement Factor H, Vitronectin, and Opticin Are Tyrosine-Sulfated Proteins of the Retinal Pigment Epithelium

**DOI:** 10.1371/journal.pone.0105409

**Published:** 2014-08-19

**Authors:** Yogita Kanan, Joseph C. Siefert, Michael Kinter, Muayyad R. Al-Ubaidi

**Affiliations:** 1 Department of Cell Biology, University of Oklahoma Health Sciences Center, Oklahoma City, Oklahoma, United States of America; 2 Department of Geriatric Medicine, University of Oklahoma Health Sciences Center, Oklahoma City, Oklahoma, United States of America; 3 Free Radical Biology and Aging Research Program, Oklahoma Medical Research Foundation, Oklahoma City, Oklahoma, United States of America; University of Florida, United States of America

## Abstract

Lack of tyrosine sulfation of ocular proteins results in disorganized photoreceptor structure and drastically reduced visual function, demonstrating the importance of this post-translational modification to vision. To understand the role that tyrosine sulfation plays in the function of ocular proteins, we identified some tyrosine-sulfated proteins in the retinal pigment epithelium using two independent methods, immuno-affinity column purification with an anti-sulfotyrosine specific antibody and computer-based sequence analysis of retinal pigment epithelium secretome by means of the prediction program Sulfinator. Radioactive labeling followed by thin layer electrophoresis revealed that three proteins, vitronectin, opticin, and complement factor H (CFH), were post-translationally modified by tyrosine sulfation. The identification of vitronectin and CFH as tyrosine-sulfated proteins is significant, since both are deposited in drusen in the eyes of patients with age-related macular degeneration (AMD). Furthermore, mutations in CFH have been determined to be a major risk factor in the development of AMD. Future studies that seek to understand the role of CFH in the development of AMD should take into account the role that tyrosine sulfation plays in the interaction of this protein with its partners, and examine whether modulating sulfation provides a potential therapeutic target.

## Introduction

Tyrosine *O* sulfation, a post-translational modification employed in higher eukaryotes [1], is catalyzed by two Type II transmembrane enzymes, tyrosylprotein sulfotransferases 1 & 2 (TPST 1 & 2). It was initially described by Bettelheim in 1954, but was later found to be a common post-translational modification [2], [3]. Tyrosine sulfation occurs in the trans-Golgi compartment and requires 3′-phosphoadenosine 5′-phosphosulfate (PAPS) as a sulfate donor for the reaction [4]. It is only observed on secreted and transmembrane proteins: nuclear and cytoplasmic proteins have not been reported to have this modification [1], [5]. However, the role of tyrosine sulfation in protein function has only recently been investigated [6]–[8].

Initial analyses of the amino acid sequences surrounding the identified sulfated tyrosines showed a predominance of acidic amino acids within 5 residues surrounding the sulfated tyrosines [9]. However, later studies showed that some tyrosine-sulfated proteins do not follow these criteria, and it is the secondary structure that may expose the tyrosine residue to a TPST to be sulfated [10].

Mouse models that lack either or both TPST enzymes exhibit distinctly different phenotypes [11]–[13]. Mice lacking both TPSTs show the most drastic phenotype of cardio-pulmonary insufficiency and subsequent death within 2 months after birth [11]. Previous studies have demonstrated that these animals also display ocular defects [14], [15]. The *Tpst1* ^−/−^ mice have reduced rod electroretinographic (ERG) responses during early development, but these normalize by postnatal day 90 [14]. However, the *Tpst2* **^−/−^ mice exhibit a non-progressive reduction in rod and cone ERG functions that persists throughout** the life of the animal [14]. Most importantly, the rod and cone ERG responses of the double knockout mice were reduced to 25% and 15% of normal littermates' levels [15].

The significant reduction in rod and cone light-evoked responses in the absence of both TPSTs underscores the role that tyrosine *O*-sulfated proteins play in vision. Previous work using the anti-sulfotyrosine antibody (PSG2) showed that tyrosine-sulfated proteins are present in different ocular tissues, including the neurosensory retina and the retinal pigment epithelium (RPE) [16], [17]. Although most of the tyrosine-sulfated proteins present in the interphotoreceptor matrix are produced locally, others originate in the RPE [17]. As a first step towards the systematic identification of tyrosine-sulfated proteins involved in sensory retina/RPE function and homeostasis, we used two approaches to isolate and characterize tyrosine *O*-sulfated proteins in the RPE. The first method involved immunoaffinity purification from cow RPE extracts using the anti-sulfotyrosine antibody PSG2, as has been established previously [18]. Several proteins were identified by mass spectrometry analysis following the affinity purification. Further analyses by ectopic expression and barium hydroxide hydrolysis confirmed that vitronectin and opticin were tyrosine-sulfated.

Affinity purification using PSG2 [19] has its own limitations, such as the co-purification of partners that interact with tyrosine-sulfated proteins that may not be tyrosine sulfated, and the lack of recognition by PSG2 due to variations in the sequences surrounding the sulfation site. Therefore, a second approach was adopted in which the secretome profile of human RPE [20] was examined for proteins associated with/causing retinal diseases listed on RetNet (https://sph.uth.edu/retnet/). While three proteins in the RPE secretome, collagen type 2, alpha 1, and complement factor H (CFH), were identified to be involved in retinal disease, CFH was the only candidate predicted to be tyrosine-sulfated by the program Sulfinator [21]. Sulfinator predicted that CFH contains 5 putative sulfated tyrosines. Subjecting it to barium hydroxide hydrolysis confirmed that, similar to vitronectin and opticin, CFH is also tyrosine sulfated. The finding that both CFH and vitronectin are sulfated, combined with the identified role of tyrosine sulfation in protein-protein interactions [6], [22]–[24], is of significance to studies of AMD since drusen, which are characteristic extracellular deposits in AMD, contain both CFH and vitronectin [25], [26]. Furthermore, some AMD cases are caused by mutations in CFH [27]–[30].

Opticin, as an extracellular matrix protein, has been shown to have anti-angiogenic properties [31], and can bind collagen [32] and retinal growth hormone (GH) in chick embryonic vitreous humor [33]. Similar to CFH and vitronectin, it would be interesting to determine in future experiments whether tyrosine sulfation modulates the interaction of opticin with collagen or GH and its anti-angiogenic properties.

## Materials and Methods

### Animal studies and ethics statement

Mouse work was performed after approval from the Institutional Animal Care and Use Committee at the University of Oklahoma Health Sciences Center (IACUC 13-001) and strictly adhered to rules and regulations set forth by the National Institute of Health Guide for the Care and Use of Laboratory Animals and the Association for Research in Vision and Ophthalmology Resolution on the Use of Animals in Research. Adult mice were euthanized using CO_2_ asphyxiation. Then, the sensory retina and RPE were harvested.

### Preparation of lysates from human donor eyes

Three human donor eyes were from 57-to-67-year-old Caucasian males that had no visual problems. Eyes were obtained from either Lions Eye Institute (Tampa, FL) or from the Illinois Eye Bank (Chicago, IL). We dissected the eyes and separated the neurosensory retina and RPE, flash froze each in liquid nitrogen, and then stored at −80°C until use. Small samples of each were homogenized in buffer A (25 mM MOPS, 100 mM NaCl, pH 7.5).

### Preparation of lysates from cow and pig eyes

Adult cow and pig eyes were obtained from Country Home Meat Slaughter House (Edmond, OK). Eyes were dissected and RPE was immediately frozen in liquid nitrogen until lysate preparation as described above. Cows were not tested for bovine spongiform encephalopathy (BSE) since they were under 30 months of age, and none of the cows in the herd in that region showed any signs of the disease.

### Processing of cow eyes for affinity purification

The RPE from four fresh independent adult cow eyes were homogenized in buffer A (above) in a dounce homogenizer. The homogenate was centrifuged at 50,000×g for 30 min at 4°C and the supernatant was collected. Protein concentrations were determined in the supernatant fraction by Bradford assay, adjusted to 4 mg/ml in wash buffer 1 (W1, 25 mM MOPS, 100 mM NaCl), and loaded on the PSG2 column.

### PSG2 affinity purification of tyrosine O sulfated proteins

About 10 mg of cow RPE supernatant was filtered using a 0.45 µm syringe filter (Millipore, Billerica, MA) and loaded onto the PSG2-Affi-Gel-10 HPLC column at a flow rate of 0.1 ml/min [18]. The column was washed successively with wash buffer 1 (W1, 25 mM MOPS, 100 mM NaCl), wash buffer 2 (W2, 25 mM MOPS, 200 mM NaCl), and wash buffer 3 (W3, 25 mM MOPS, 400 mM NaCl) at a flow rate of 0.2 ml/min, then eluted with elution buffer (EB, 25 mM MOPS, 400 mM NaCl, 4 mM sulfated pentapeptide). The entire run was monitored by recording absorbance at 280 nm. The eluted samples were concentrated with acetone precipitation and fractionated by SDS-PAGE. The tyrosine-sulfated pentapeptide LDY^S^DF was synthesized (Bio-Synthesis Inc., Lewisville, TX).

### Mass spectrometry

Column fractions were separated by SDS-PAGE and the gel lane was cut into 1 mm slices. Each slice was reduced, alkylated, and subjected to in-gel trypsin digestion. The samples were analyzed using a Thermo Scientific LTQ-XL linear ion trap system with an Eksigent splitless nanoflow HPLC. Ten µL volumes of each digest were injected. A data-dependent analysis acquired one mass spectrum and 9 collision induced dissociation (CID) spectra per cycle. The CID spectra were used to search the cow RefSeq database using the program Mascot. All identified proteins exceeded a minimal identification criteria of at least 2 CID spectra matching unique peptide sequences with ion scores greater than 50. Since at this time only secreted or transmembrane proteins have been identified as tyrosine-sulfated [1], [5], only these proteins were included in Tables 1 & 2. Nuclear and cytoplasmic proteins that co-purified in the column were therefore eliminated.

**Table 1:** List of potential tyrosine-sulfated proteins in cow RPE.

	Protein name	Mascot Score	RefSeq#	Coverage %
*	Opticin	605	45429965	26
*	Lumican	630	27806853	31
*	Vitronectin	313	78045497	15
*	Heparin cofactor II	722	76639676	26
*	Similar to Amyloid beta A4 protein precursor	851	76607645	22
1.	Fibrinogen, gamma	1093	27806893	43
2.	Complement C4	978	76650940	10
3.	Dickkopf related protein 3	863	76635678	28
4.	Fibulin 2	774	76649536	13
5.	Fibrinogen beta	657	76638241	49
6.	Secreted frizzled related protein	662	27806625	39
7.	Tubby like protein 1	557	76672237	54
8.	Spondin 1	480	27807443	14
9.	Retinol Binding protein 3 (IRBP)	467	27806445	9
10.	Fibroblast growth factor	381	27806627	31
11.	Fibrinogen alpha	319	75812954	10

Sixteen proteins were identified in cow RPE by MALDI-MS analysis of SDS-PAGE gel slices of PSG2 immunoaffinity column eluent. The asterisks mark those proteins at 75 and 50 kD, shown in Figure 2C. NCBI reference sequence database ID numbers (RefSeq#) and percent sequence coverage (coverage %) are also indicated for each protein. Inclusion in this table does not confirm that the protein is tyrosine-sulfated. Some of the proteins may be isolated by the affinity column as a result of their interaction with sulfated proteins.

**Table 2:** List of potential tyrosines that may be sulfated on human and cow vitronectin, opticin, and CFH, as identified by Sulfinator.

Name	Site	Sequence	Reference for Tyrosine sulfation
	75*	MPEDE**Y**TVYDD	
	78*	DEYTV**Y**DDGEE	
Human Vitronectin	282	KQYWE**Y**QFQHR	Yu Y, Hoffhines AJ, Moore KL, Leary JA (2007) Determination of the sites of tyrosine O-sulfation in peptides and proteins. Nat Methods 4: 583–588.
	417	LGANN**Y**DDYRM	
	420	NNYDD**Y**RMDWL	
Cow	75	LPEDE**Y**GFHDY	Novel
Vitronectin	80	YGFHD**Y**SDAQT	
Human Opticin	65	IDLSN**Y**EELTD	Novel
	71	EELTD**Y**GDQLP	
Cow Opticin	61	DELID**Y**GDQLP	Novel
Human CFH	243	NMGYE**Y**SERGD	
	534	NDTLD**Y**ECHDY	
	709	LSSPP**Y**YYGDS	Novel
	710	SSPPY**Y**YGDSV	
	711	SPPYY**Y**GDSVE	
Cow CFH	168	EPDQE**Y**TYGQV	Novel
	170	DQEYT**Y**GQVVQ	
	465	ESTFT**Y**PLNKQ	
	473	NKQTE**Y**KCKPG	
	575	PEMDP**Y**LNAYP	
	579	PYLNA**Y**PRKET	
	585	PRKET**Y**KVGDV	

Those sites that have been experimentally proven are marked by an asterisks and citation is provided.

### RPE secretome analysis

Members of the RPE secretome [20] were screened for disease-causing proteins listed in the Retinal Information Network (RetNet). The candidate proteins that caused disease were then plugged into the tyrosine sulfation prediction program Sulfinator [21] to predict their tyrosine sulfation status.

### Immunoprecipitation and immunoblotting

Immunoprecipitation and immunoblotting were performed according to previously published methods [17], [34]. The following antibodies were used: anti-vitronectin antibody (Santa Cruz Biotechnology Inc., Dallas, TX), anti-opticin antibody (Santa Cruz Biotechnology Inc.), and anti-CFH antibody (AbD Serotec, Raleigh, NC). The purified human plasma CFH was obtained from AbD Serotec.

### Metabolic labeling, barium hydroxide hydrolysis, and thin layer electrophoresis analysis

HEK 293T cells were independently transiently transfected with recombinant human vitronectin (VTN, Genecopoeia, Rockville, MD), recombinant myc-tagged human Opticin (OPTC, Genecopeia), or human recombinant complement factor H (CFH, Genecopoeia). Eight hours after transfection, media was replaced with sulfate-free Joklik-modified Eagle's media (Sigma, St. Louis, MO) containing 2% dialyzed fetal bovine serum. To this media, 0.15 mCi/ml of Na_2_^35^SO_4_ (Perkin Elmer, Waltham, MA) was added. About 48 hours later, the media was harvested, fractionated by SDS-PAGE, and transferred onto polyvinylidene difluoride (PVDF) membranes (Millipore, Billerica, MA). The radioactive bands were cut out and subjected to barium hydroxide hydrolysis and thin layer electrophoresis (TLE) according to published methods [11], [18].

### Deglycosylation assays

CFH was treated for 2 hours at 37°C with PNGase F, according to the manufacturer's instruction (NEB BioLabs, Ipswich, MA).

## Results

### RPE extracts are a rich reservoir of tyrosine-sulfated proteins

**Figure 1:**
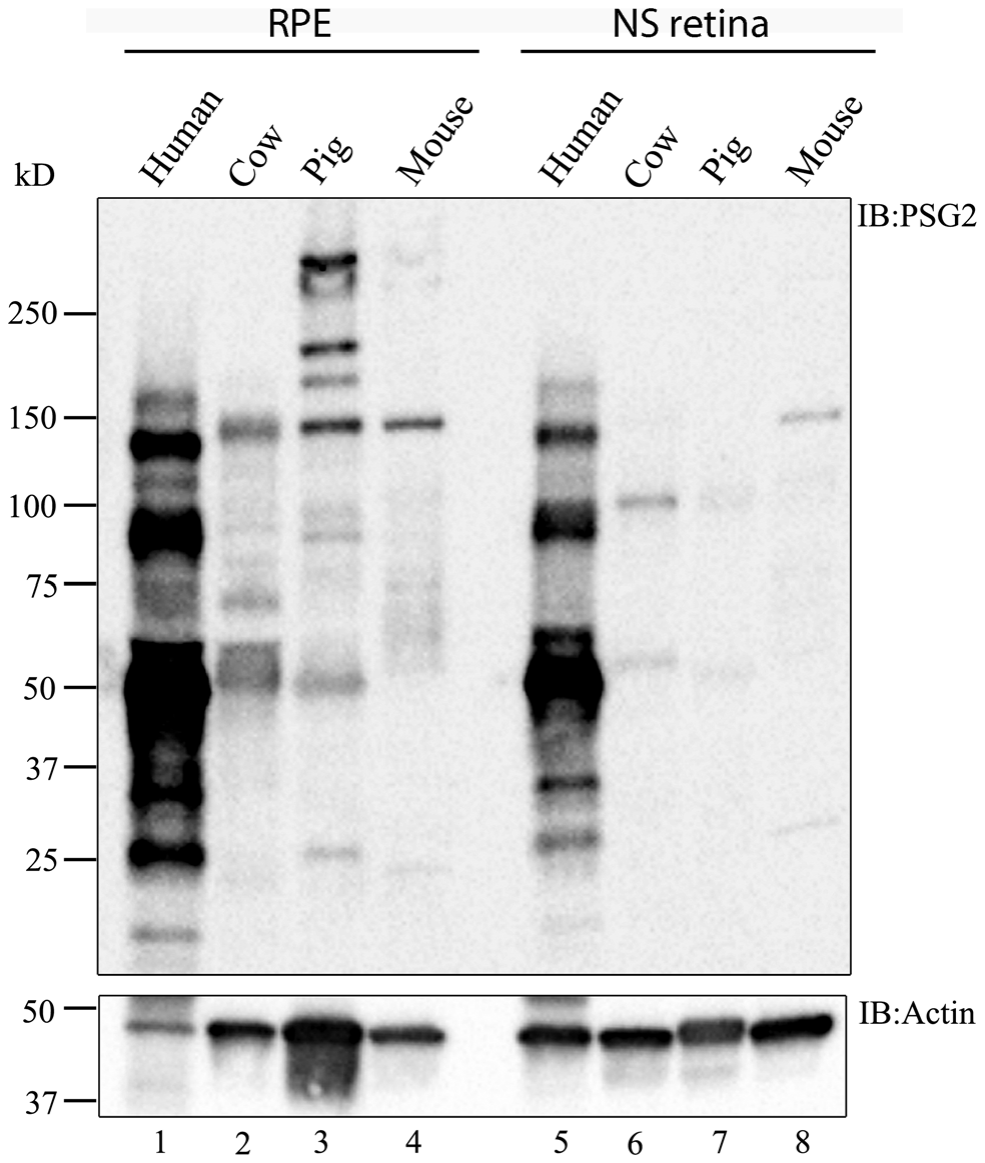
Immunoblot analysis of neurosensory (NS) retinal and RPE extracts to identify tyrosine-sulfated proteins. Immunoblot analysis of 50 µg of RPE (lanes 1–4) and neurosensory retinal extracts (lanes 5–8) from human, cow, pig, and mouse RPE probed with the anti-sulfotyrosine antibody PSG2. Blots were repeated 3 independent times using biologically different samples.

Tyrosine-sulfated proteins have been previously shown to be expressed in the neurosensory retina and RPE [16], [17]. Furthermore, some of the sulfated proteins that were present in the neurosensory retina originated in the RPE [17]. To determine the differential distribution of tyrosine-sulfated proteins in the neurosensory retina and RPE, immunoblot analyses of mouse, pig, cow, and human neurosensory retinal and RPE lysates were performed and probed with PSG2. The analysis revealed that the RPE harbors a relatively higher number of tyrosine-sulfated proteins than the neurosensory retina in each of the four mammalian species tested (Figure 1). The sizes of sulfated proteins in the RPE ranged from 20 kD to >250 kD. While some sulfated protein bands appeared to be conserved across species tested, other tyrosine-sulfated protein bands seemed to be species-specific. An across-species conserved sulfated protein may appear at different sizes on immunoblots because tyrosine-sulfated proteins are secreted proteins and, therefore, may be differentially glycosylated [35], [36] due to species-specific glycosylation patterns. The tyrosine-sulfated proteins in human lysates appear more prominent when compared to those in other species. This may be partly due to the fact that PSG2 was raised against the tyrosine-sulfated N terminus of human PSGL-1 [19], and hence may better recognize human tyrosine-sulfated proteins. Alternatively, since recognition by PSG2 was shown to be context-dependent [19], better recognition of human proteins may also have to do with the environment surrounding the sulfated tyrosines.

### Immunoaffinity purification of tyrosine-sulfated proteins from the RPE

**Figure 2:**
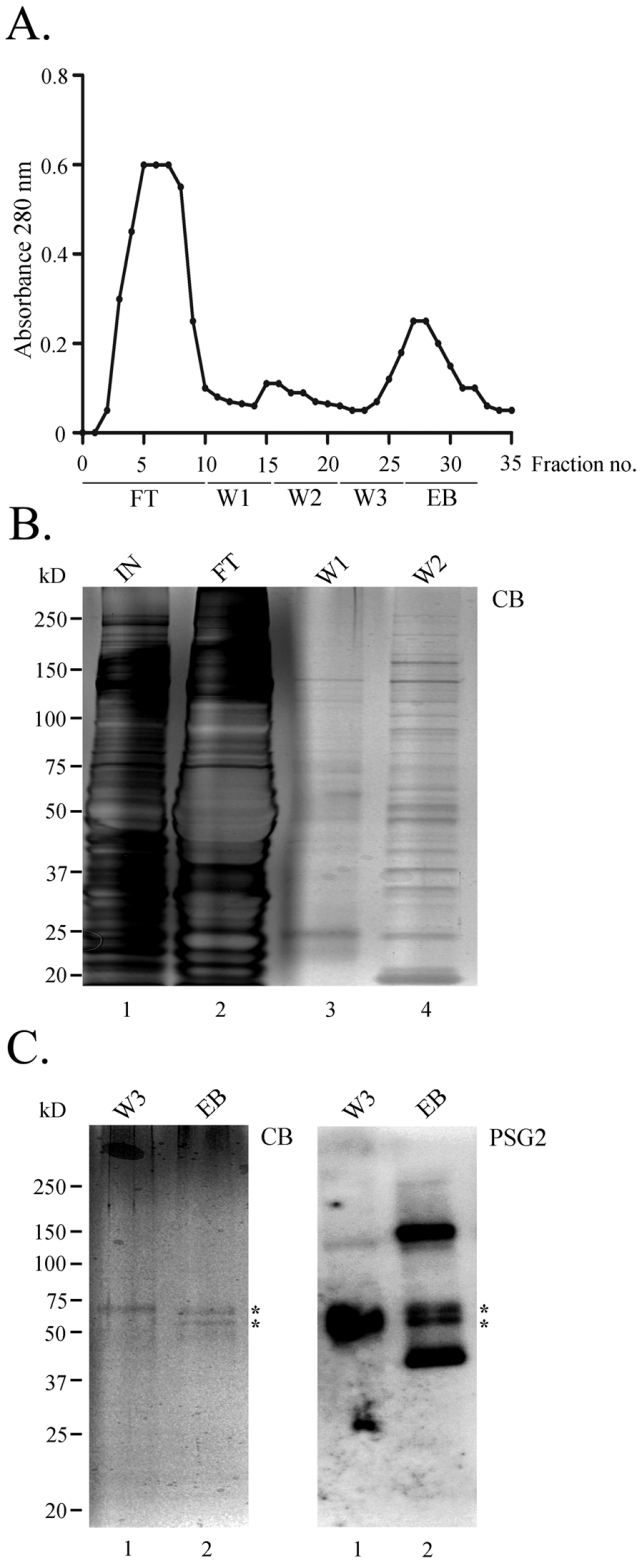
PSG2-immunoaffinity column purification of tyrosine-sulfated proteins from cow RPE. (A). The elution profile was monitored by following absorbance at 280 nm. Following loading, the column was washed with buffers W1, W2, and W3. Elution was performed in buffer W3 containing 4 mM sulfated pentapeptide (EB). (B). Twenty-six microliter aliquots from input (IN), flow-through (FT), wash 1 (W1), and wash 2 (W2) were fractionated by SDS-PAGE, and proteins were visualized by staining with Coomassie blue dye. (C). Left, SDS-PAGE of 26 µL of wash 3 (W3) and eluted samples (EB) from the immunoaffinity column stained with Coomassie blue (CB) and right, immunoblotted with PSG2. Asterisks indicate the bands that were prominent on Coomassie blue-stained gel (CB) and were also recognized by PSG2 as tyrosine-sulfated.

We chose to use cow RPE extracts in the affinity purification with PSG2 because RPE lysates contain more tyrosine-sulfated proteins than the neurosensory retina, most of the sulfated proteins that appear in human RPE are also observed in cow RPE, and it was easy to obtain a sufficient number of independent samples. Monitoring the column flow-through by measuring absorbance at 280 nm showed that most of the unbound proteins passed through the column in the flow-through (FT) fraction (Figure 2A). The remaining nonspecifically bound proteins were washed off the column with increasing salt washes (W1, W2 & W3, Figure 2A), while the potential tyrosine-sulfated proteins were eluted with EB containing 4 mM sulfated pentapeptide (EB, Figure 2A).

Proteins in the FT, W1, W2, W3, and EB fractions were separated by SDS-PAGE, followed by Coomassie blue staining of the gel. Comparing equal volume aliquots from each fraction confirmed the column absorbance results and showed that the majority of the non-specific proteins were already cleared away by W2 (compare W2 and W3, Figure 2B&C). The EB fraction contained two enriched bands (asterisks, Figure 2C, CB) visible after Coomassie blue staining of the gel. PSG2-immunoblot analysis of EB showed that the two enriched bands were tyrosine-sulfated proteins: it is important to note that there were other tyrosine-sulfated proteins in this fraction that were not obvious by Coomassie blue staining (Figure 2C, PSG2). Interestingly, the upper asterisk-marked band in Figure 2C was also observed in W3, and was also sulfated, as shown after probing with PSG2 (W3&EB, Figure 2C). While only 20% of the peptide eluted fraction was run on the SDS-PAGE, the remaining 80% was acetone-precipitated and fractionated by SDS-PAGE for 20 minutes to remove the pentapeptide. The entire lane was sliced into multiple 1 mm portions and subjected to in-gel trypsin digestion, followed by LC-tandem MS analysis. Table 1 presents the identity of 16 proteins that were selectively eluted from the column and identified by MS/MS. Among the identified proteins, lumican, heparin cofactor II, complement C4, amyloid beta protein, fibulin 2, and fibrinogen (α, β, and γ) have been previously identified to be tyrosine-sulfated and their sites of sulfation identified [37]–[43]. Retinol binding protein 3, Spondin 1, basic fibroblast growth factor, secreted frizzled related protein, and Tubby-like protein 1 have not previously been shown to be tyrosine-sulfated. It is important to mention that there is no obvious functional relationship between the identified proteins, since they belong to different classes of extracellular proteins. For example, retinol binding protein is involved in the retinoid cycle [44], dickkopf-related protein is involved in a signaling pathway [45], and fibrinogen is involved in the blood clotting pathway [46].

Due to the myriad functions performed by vitronectin, and since opticin has recently been shown to exert anti-angiogenic properties, we chose to focus on vitronectin and opticin. However, the unavailability of anti-bovine specific antibodies capable of immunoprecipitating vitronectin and opticin from bovine RPE limited our studies to human proteins instead. While vitronectin has previously been shown to be tyrosine-sulfated in human hepatoma-derived cell line Hep G2 and human plasma [47], [48], its tyrosine sulfation status in the human RPE is unknown. Furthermore, the tyrosine-sulfation of opticin has not been demonstrated.

### Vitronectin is tyrosine-sulfated in human RPE

**Figure 3:**
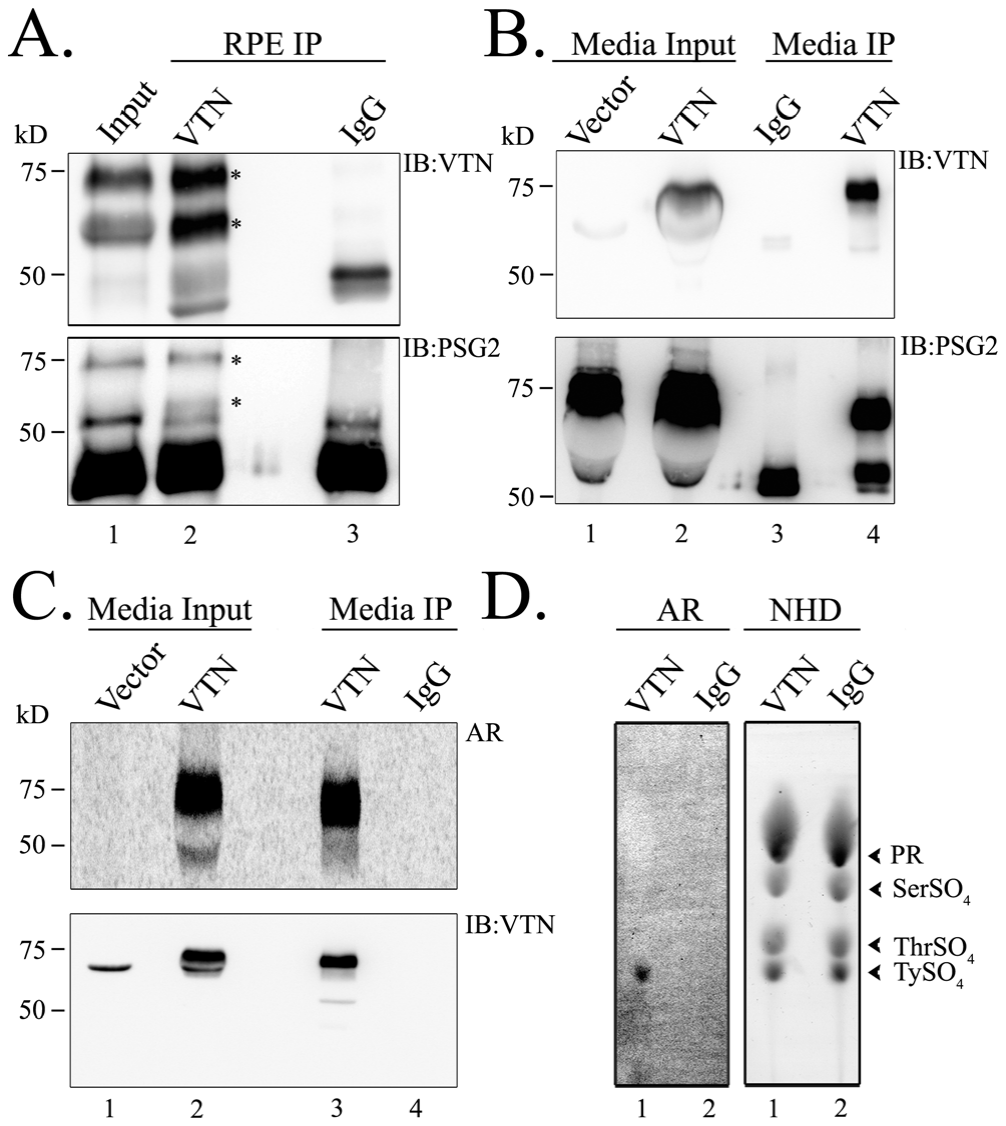
Native human RPE vitronectin is tyrosine-sulfated. Vitronectin was immunoprecipitated from 500 µg human RPE lysates using anti-VTN antibody (lane 2) or mouse IgG (lane 3). Immunoprecipitants were fractionated by SDS-PAGE, transferred, and immunoblotted using anti-VTN antibody or anti-sulfotyrosine PSG2 antibody. Immunoprecipitation and western blots were repeated 3 independent times using biologically different human RPE samples. (B). Ectopically-expressed vitronectin is tyrosine-sulfated. Recombinant VTN or empty vector (pcDNA3.1) were transfected into HEK 293T cells and immunoprecipitated from conditioned media using anti-VTN antibody (lane 4) or mouse IgG (lane 3). Immunoprecipitants were electrophoresed and immunoblotted using anti-VTN antibody or anti-sulfotyrosine PSG2 antibody. Immunoprecipitation and western blots were repeated 3 independent times after independent VTN transfections. (C). ^35^S-metabolic labeling of recombinant vitronectin *in vitro*. Vitronectin-transfectants were radiolabelled with ^35^Sulfate. Following radiolabeling, vitronectin was immunoprecipitated and blots were either subjected to autoradiography (AR) or immunoblotted with anti-VTN antibody. (D). Radiolabeled vitronectin bands were excised from the membrane along with equivalent areas from mouse IgG immunoprecipitants, and alkaline hydrolysis was performed. The samples were then spiked with sulfo-amino standards tyrosine sulfate, threonine sulfate, and serine sulfate, and subjected to thin layer electrophoresis (TLE) on cellulose plates. Following TLE analysis, sulfo-amino standards (NHD) were visualized either by spraying with Ninhydrin or autoradiography (AR). TLE experiments were repeated at least three independent times.

To confirm that human vitronectin is tyrosine-sulfated *in vivo*, we immunoprecipitated it from human RPE lysates using anti-vitronectin antibody. Vitronectin in human RPE was observed to migrate as two bands of sizes 65 kD and 75 kD (lane 1, Figure 3A). These two isoforms have been detected in multiple tissues [49]–[51]. It has been shown that the 65 kD band results from the cleavage of the 75 kD vitronectin band by the protease furin [51]. Both the 65 kD and 75 kD isoforms, detected in the RPE input, were immunoprecipitated by the anti-vitronectin antibody (asterisks, Figure 3A, upper panel), but not by mouse IgG. Additionally, using PSG2, we detected that both isoforms were tyrosine-sulfated (Figure 3A, lower panel).

While the PSG2 antibody identifies VTN as a tyrosine-sulfated protein, the gold standard for classifying a protein as tyrosine-sulfated is the actual detection of sulfated tyrosines in the protein by barium hydroxide hydrolysis. To accomplish that, recombinant vitronectin was expressed in a heterologous system in the presence of radioactive sulfate, followed by barium hydroxide hydrolysis and thin layer electrophoresis (TLE) in the presence of a non-radioactive sulfated tyrosine standard. The co-localization of the radiolabelled sulfated tyrosine from the recombinant protein with the non-radioactive sulfated tyrosine standard on the TLE plate confirms that the protein of interest is subjected to tyrosine sulfation. To this purpose, recombinant human vitronectin and a control vector (pcDNA 3.1) were transfected into HEK293T cells. Since vitronectin is a secreted protein, media was collected and immunoblotted with anti-vitronectin antibody. Interestingly, only the 75 kD isoform was expressed in cells transfected with recombinant vitronectin (Figure 3B). The sole presence of the 75 kD isoform may be due to the absence of furin in HEK293T cells. The transfected 75 kD vitronectin isoform was immunoprecipitated from the media and shown by PSG2 to be tyrosine-sulfated (Figure 3B). The recombinant vitronectin was then metabolically labeled with ^35^S followed by immunoprecipitation and autoradiography to confirm the radiolabeling of the protein (Figure 3C, AR). The radioactive 75 kD band was confirmed to be vitronectin by the anti-vitronectin antibody (Figure 3C). The radiolabeled vitronectin band and a similar-sized membrane from an equivalent region of the IgG control lane were cut from the membrane and subjected to alkaline hydrolysis using the barium hydroxide method described by Huttner et al [2]. The co-localization of the radioactive tyrosine-sulfate from the radiolabelled vitronectin hydrolysates (Figure 3D) with the non-radioactive tyrosine-sulfate standard detected after ninhydrin staining of the TLE plate (Figure 3D) confirmed that human vitronectin is tyrosine-sulfated.

### Opticin is tyrosine-sulfated in vivo

**Figure 4:**
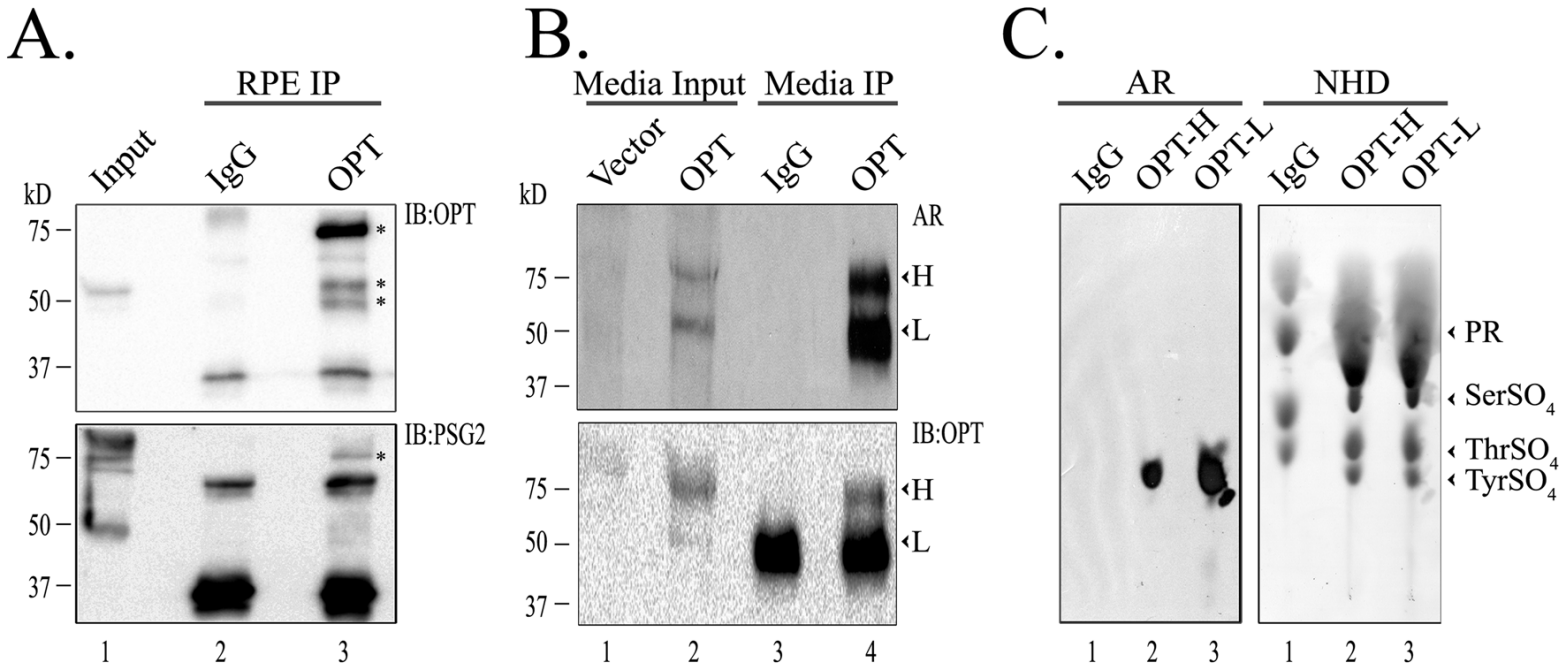
Tyrosine sulfation of ectopically expressed opticin. (A). Opticin was immunoprecipitated from 500 µg human RPE lysates using anti-OPT antibody (lane 3) or mouse IgG (lane 2). Immunoprecipitants were electrophoresed and immunoblotted using anti-OPT antibody (upper panel) or PSG2 (lower panel). Immunoprecipitation and Western blots were repeated 3 independent times using biologically different human RPE samples. (B). Metabolic labeling of recombinant opticin. Recombinant opticin or empty vectors were transfected into HEK293T cells and cells grown in presence of radioactive sulfate. Following radiolabeling, opticin isoforms were immunoprecipitated using anti-OPT antibody (lane 4) or mouse IgG (lane 3). The blots were then subjected to autoradiography (AR) and immunoblotted for opticin. The 65 kD and 55 kD isoforms are depicted as ‘H’ and ‘L’, respectively. Immunoprecipitation and western blots were repeated 3 independent times after independent OPT transfections. (C). Both the 65 kD ‘H’ and 55 kD ‘L’ isoforms were excised from the blot, along with equivalent areas around 55 kD from mouse IgG immunoprecipitants, and analyzed by barium hydroxide hydrolysis. The samples were then spiked with a mixture of tyrosine sulfate, threonine sulfate, and serine sulfate as standards, and subjected to thin layer electrophoresis (TLE) on cellulose plates. Following TLE analysis, the plate was sprayed with ninhydrin to visualize the sulfo-amino standards (NHD) and was autoradiographed (AR). TLE experiments were repeated at least three independent times.

Opticin was identified in human RPE as a 55 kD protein (lane 1, Figure 4A). Since this size is similar to the heavy chain of the antibody, which is also tyrosine-sulfated [52], opticin was immunoprecipitated using anti-opticin antibody and eluted under non-reducing conditions, so that the tyrosine-sulfated heavy chain of the antibody would not mask the opticin isoform. The anti-opticin antibody was successful in bringing down the 55 kD monomeric opticin band as well as multiple aggregated opticin bands of sizes 55–75 kD, with the 75 kD band being the most abundant (asterisks, Figure 4A). Opticin was not pulled down with the mouse IgG antibody (Figure 4A, upper panel). A companion blot probed with PSG2 showed tyrosine sulfation recognition by the antibody of the 75 kD aggregated opticin band (asterisk, Figure 4A, lower panel).

We then expressed recombinant opticin ectopically in HEK293T cells and metabolically labelled the protein with ^35^S. This method provides an advantage over cold experiments since it allow us to differentiate between ^35^S-opticin and the non-radiolabeled IgG heavy chain, even under reduced conditions. After radiolabeling, two isoforms (55 kD and 65 kD) were found to have incorporated the radiolabel only in the conditioned media of transfected cells (Figure 4B). It is worth mentioning that the two isoforms of opticin observed here have been reported by other groups [53]–[55]. Opticin has also been shown to be O-glycosylated and, therefore, the two isoforms may be differentially O-glycosylated isoforms [56]. The two isoforms were immunoprecipitated (lane 4, Figure 4B) and visualized after autoradiography. These bands are identified in Figure 4B as ‘lower band (L)’ for the 55 kD protein and ‘higher band (H)’ for the 65 kD protein.

To verify that the radiolabelled bands were opticin, after autoradiography we blotted the bands with anti- opticin antibody, which recognized both the L and the H isoforms of opticin in the media of transfected cells (Figure 4B). However, in the anti-opticin immunoprecipitated lane (lane 4, Figure 4B), only the 65 kD (H) opticin band is distinct, because the heavy chain IgG band from the antibody masks the L opticin isoform under reducing conditions (Figure 4B). Finally, we performed barium hydroxide hydrolysis to confirm that the radiolabelled isoforms were tyrosine-sulfated. Again, the co-migration of the radioactive tyrosine-sulfate from the radioactive protein with the non-radioactive tyrosine-sulfate standard on TLE plate confirms that opticin contains sulfated tyrosines (Figure 4C).

### Analysis of RPE secretome identifies CFH as a potentially sulfated protein

Parallel to the immunoaffinity purification of tyrosine-sulfated proteins from the RPE, we performed an independent analysis on the human RPE secretome [20]. Two criteria were chosen for this analysis. The first was to select members of the RPE secretome in which mutations had been identified to be associated with human visual disorders according to the Retinal Information Network (RetNet). The second criterion was to determine which of these selected proteins is predicted to be tyrosine-sulfated by Sulfinator [21].

Our analysis of the human RPE secretome yielded two tyrosine-sulfated proteins, complement factor H (CFH) and collagen type 2 (alpha 1), which are implicated in AMD and Stickler syndrome, respectively [27], [57]–[59]. Analyses by Sulfinator predicted only CFH as a tyrosine-sulfated protein, with 5 possible sites at tyrosines 243, 534, 709, 710, and 711 (Table 2). In addition, human vitronectin and opticin were similarly analyzed by Sulfinator and were found to be tyrosine-sulfated (Table 2). Human vitronectin was predicted to contain five sulfated tyrosines at 75, 78, 282, 417, and 420. Two of these residues, 75 and 78, have been confirmed to be sulfated [48]. Finally, human opticin was predicted to contain two tyrosine-sulfated residues at positions 65 and 71 (Table 2). It is interesting to note that while vitronectin and opticin were originally identified using immunoaffinity purification from bovine extracts, Sulfinator also predicts bovine opticin and vitronectin to be tyrosine-sulfated (Table 2). In addition, bovine CFH was predicted to be tyrosine-sulfated by sequence analysis (Table 2). This suggests the exciting possibility that protein tyrosine sulfation is likely conserved across species.

### Complement factor H is tyrosine-sulfated in its native form

**Figure 5:**
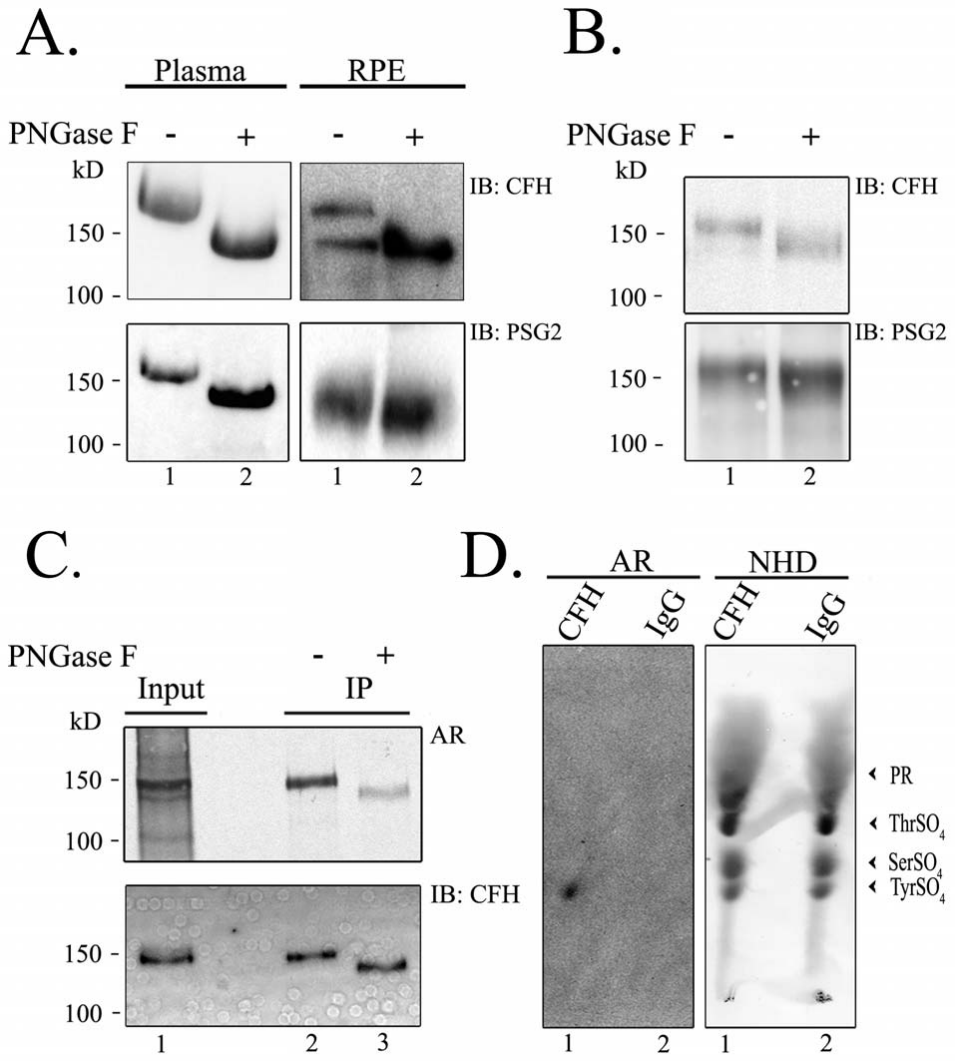
Native human plasma and RPE CFH is sulfated. (A) About 4 µg of purified human plasma CFH was either untreated (lane 1) or treated with PNGase F (lane 2), and then immunoblotted with anti-CFH antibody or with PSG2. CFH was also immunoprecipitated from human RPE, and was either untreated (lane 1) or treated with PNGase F (lane 2), and then immunoblotted with anti-CFH antibody or with PSG2. Western blots were repeated 3 independent times from human plasma and RPE samples. (B) A recombinant human CFH clone was transfected into HEK293T cells, immunoprecipitated with anti-CFH antibody, and was either directly electrophoresed (lane 1) or first treated with PNGase F (lane 2). The immunoprecipitants were immunoblotted with anti-CFH antibody or with PSG2. Immunoprecipitation and Western blots were repeated 3 independent times after CFH transfections. (C). Recombinant CFH was transfected into HEK293T cells and radiolabelled with radioactive sulfate, then immunoprecipitated and subjected to PNGase treatment (lane 3) or left untreated (lane 2). The blots were autoradiographed (AR), then immunoblotted with anti-CFH antibody. (D). Radiolabelled CFH was analyzed by barium hydroxide hydrolysis and autoradiographed (AR). TLE experiments were repeated at least three independent times.

CFH has been shown to be mainly produced in the liver and secreted into the plasma [60], [61], and secreted by the RPE in ocular tissue [28], [62]. To demonstrate that CFH is tyrosine-sulfated *in vivo*, purified human plasma CFH was fractionated by SDS-PAGE and immunoblotted with anti-CFH and PSG2 antibodies. Immunoblot analysis with PSG2 identified human CFH as tyrosine-sulfated (Figure 5A). Since CFH has eight N-glycosylated residues [63], deglycosylation of the protein with PNGase F eliminated the glycosidic residues and decreased the size of the protein by ∼18 kD [63]. This glycosidic elimination enhanced detection by PSG2 (Figure 5A). The same strategy was adopted to human RPE immunoprecipitated CFH. Human RPE CFH showed the presence of 2 CFH bands, a glycosylated and non-(or partially)-glycosylated isoform (Figure 5A, IB:CFH), even in the absence of deglycosylation with PNGase F. However, PNGase F treatment of the immunoprecipitated CFH collapsed the two bands into one non-glycosylated CFH band (Figure 5A, IB: CFH). Both of the non-glycosylated bands were recognized by PSG2 in the presence and absence of PNGase F (Figure 5A, IB: PSG2). Interestingly, PSG2 showed reactivity only to the native non-glycosylated isoform of CFH, not the glycosylated endogenous CFH (lower right panel of Figure 5A, IB: PSG2).

The same deglycosylation strategy was used in *in vitro* studies. Recombinant CFH was immunoprecipitated from the media of human CFH-transfected HEK293T cells. One portion was subjected to PNGase F treatment while another was left untreated. Immunoblot analysis showed a size decrease with PNGase F treatment (Figure 5B). Both the PNGase F-treated and untreated immunoprecipitants were recognized by PSG2, with the PSG2 showing better reactivity to the PNGase F- treated immunoprecipitant (compare lane 2 to 1, Figure 5B).

*In vitro* metabolic labeling with ^35^S showed that CFH incorporated the label (Figure 5C). PNGase F treatment eliminated ∼75% of ^35^S radioactivity as determined by densitometry (Figure 5C). This suggests that the N-glycosylated residues on CFH are also sulfated, which is in agreement with a previous report [64]. Barium hydroxide hydrolysis and TLE showed that the remaining ∼25% of label on CFH is on tyrosine(s), as indicated by the co-localization of the radioactive tyrosine sulfate from radioactive CFH with the non-radioactive tyrosine sulfate standard (Figure 5D).

## Discussion

Previous studies using the *Tpst1^-/-^*, *Tpst2^-/-^*, and double knockout mouse models demonstrated the importance of tyrosine sulfation for vision. Since proper visual transduction results from a hemostatic relationship between two closely associated tissues, the neurosensory retina and the RPE, we compared the abundance of tyrosine-sulfated proteins in these two tissues. Immunoblot analysis with PSG2 showed a greater abundance of tyrosine-sulfated proteins in the RPE compared to the neurosensory retina, across species. To identify tyrosine-sulfated proteins in the RPE, two approaches were used. In the first approach, we used immunoaffinity purification with PSG2 antibody to isolate the tyrosine-sulfated proteins. The second method involved applying the tyrosine sulfate prediction program Sulfinator to analyze proteins in the human RPE secretome to select candidate protein(s) that are involved in human disease, as well as potentially tyrosine-sulfated.

Vitronectin and opticin were identified using the first approach, and complement factor H (CFH) was identified as a possible sulfated protein using the second approach. To confirm that these three proteins are tyrosine-sulfated, multiple methods were adapted. A non-radioactive scheme involved immunoprecipitating the said protein with antigen-specific antibodies, followed by immunoblotting with anti-sulfotyrosine antibody PSG2. A radioactive method included metabolically labeling the protein, and performing immunoprecipitation followed by barium hydroxide analysis and thin layer electrophoresis. The barium hydroxide method is the gold standard for confirming tyrosine sulfation because it hydrolyzes the carbohydrate moieties on a protein and has the ability to determine whether the sulfate is on a tyrosine or on a carbohydrate moiety [2].

Opticin was isolated following immunoaffinity purification with PSG2. Further analysis by immunoblotting with PSG2 and barium hydroxide hydrolysis confirmed that it was tyrosine-sulfated. Opticin is an extracellular matrix protein that has recently been shown to have anti-angiogenic properties *in vivo* and *in vitro*. *In vivo* comparisons of wild-type and opticin knockout animals in the oxygen-induced retinopathy model of neovascularization showed more neovascularization in the knockout animal [31]. These results were further examined *in vitro*, in which it was shown that opticin binds collagen and thereby inhibits endothelial cell integrins α(1)β(1) and α(2)β(1) from binding collagen, a necessity for pro-angiogenic signaling [32]. It has also been shown that opticin can bind retinal growth hormone (GH) in chick embryonic vitreous humor [33]. It would be interesting to see if removing sulfation on opticin modulates its interaction with collagen or GH and if it affects anti-angiogenic functions.

Vitronectin has been previously shown to be tyrosine-sulfated in human plasma [48]. Its tyrosine-sulfated residues were identified as Tyr-75 and Tyr-78 [48]. These two residues are close to the ‘RGD’ cell attachment site on the protein, which resides between residues 64–66. The RGD sites on vitronectin have been previously shown to bind integrin receptor αvβ3 and αvβ5 [52], [65]. However, for this to occur, the ‘RGD’ site must be exposed to the surface, which can be influenced by type of surrounding residues. For example, the presence of a proline residue that follows the ‘RGD’ site silences the motif by preventing surface accessibility [66]. Therefore, the function of tyrosine sulfation, due to its hydrophilic nature and close proximity to the ‘RGD’ site, may be to expose the RGD domain at the surface of the protein, facilitating its interaction with integrin receptors on cells. Since tyrosine sulfation has been shown to be necessary for protein-protein interactions [6], [22]–[24], the presence of a highly charged sulfate group may facilitate the interaction of vitronectin with a positively charged domain(s) on an interacting partner. As the RGD-Integrin attachment influences many functions such as cell migration, adhesion, growth, and differentiation [67], [68], the presence of tyrosine sulfation may potentially modulate all of these functions.

Another function of vitronectin is complement regulation. Vitronectin inhibits the complement cascade by binding the membrane attack complex (MAC), the final product in the complement cascade [69], and therefore protects cells against complement attack. Complement damage in the eye is a leading cause of vision loss and causes AMD [70]–[72], which results in the increased presence of drusen in Bruch's membrane [73]. A major component of drusen is vitronectin and complement proteins [25]. It has previously been shown that vitronectin is upregulated during complement attack on RPE cells [74], [75]. Since vitronectin is a tyrosine-sulfated protein, it would be interesting to study if eliminating tyrosine sulfation decreases its binding to the MAC complex and results in its inability to inhibit the complement cascade, therefore exacerbating complement damage to cells. Future studies in which tyrosine-sulfated sites on vitronectin are mutated to phenylalanines will address the role of tyrosine sulfation in MAC binding.

Sulfinator analysis of the RPE secretome predicted CFH as a tyrosine-sulfated protein. This protein is produced by the RPE [20], [62] and is also involved in complement regulation. CFH inhibits complement activation by acting as a cofactor in Factor I-mediated decay of C3-covertase [76]. Mutations in CFH have been implicated in AMD [27], [29], [30]. In addition to binding C3 convertase, CFH self-interacts [81], [82] and has also been shown to bind multiple proteins in the complement system, such as C-reactive protein (CRP) [77], [78], C3b [79], and C3d [80,80]. Since the main function of tyrosine sulfation is modulation of protein-protein interaction [6], [22]–[24], the function of tyrosine sulfation in CFH may be to influence the interactions between some of these complement proteins and to inhibit complement activation. To test this possibility, future studies will focus on the identification of the sulfated tyrosine(s) on CFH using site-directed mutagenesis to phenylalanines, followed by functional studies to examine if the interaction of the protein with its binding partner is affected. Ultimately, we would generate knock-in mice that have the endogenous wild-type sulfated tyrosine(s) replaced with phenylalanine(s). This mouse will be the ideal model with which to better understand the role that tyrosine sulfation plays in the function of CFH in complement regulation.

Due to the involvement of tyrosine sulfation in protein-protein interactions [6], , using the PSG2 immunoaffinity column to isolate tyrosine *O*-sulfated proteins would not only pull down tyrosine-sulfated proteins, but also nonsulfated proteins which may be co-purifying due to their direct or indirect interaction with tyrosine-sulfated proteins. Therefore, each protein isolated from the column needs to be independently verified as either a tyrosine-sulfated protein or an interacting protein.

In conclusion, this report identifies the tyrosine-sulfated proteins present in the RPE. Since tyrosine sulfation can influence protein-protein interactions, any future studies of vitronectin, opticin, or CFH should involve identifying the tyrosine-sulfation sites on these proteins. This will be followed by functional studies to examine if the interaction of the protein with its binding partner is affected.
